# Associations of TNF-α, Uric Acid, and the Leptin/Adiponectin Ratio with HOMA-IR in Hospitalized Adults: A Cross-Sectional Secondary Analysis of 147 Patients

**DOI:** 10.3390/medicina62101825

**Published:** 2026-09-22

**Authors:** Alina Andreea Tischer, Loredana Gabriela Stana, Ovidiu Calin Ilie, Adrian-Cosmin Ilie, Lavinia Craciun, Ionut Capraru, Emilia Elena Clej

**Affiliations:** 1 Ear-Nose-Throat Department, “Victor Babeș” University of Medicine and Pharmacy, 300041 Timișoara, Romania; tischer.alina@umft.ro; 2 Department I, Discipline of Anatomy and Embryology, Faculty of Medicine, “Victor Babeș” University of Medicine and Pharmacy, Eftimie Murgu Square 2, 300041 Timișoara, Romania; loredana.stana@umft.ro (L.G.S.); craciun.lavinia@umft.ro (L.C.); 3 Department I, Discipline of Dental Prostheses Technology (Dental Technology), Faculty of Dental Medicine, “Victor Babeș” University of Medicine and Pharmacy, 300041 Timișoara, Romania; 4 Department of Public Health and Sanitary Management, “Victor Babeș” University of Medicine and Pharmacy, Eftimie Murgu Square 2, 300041 Timișoara, Romania; ilie.adrian@umft.ro; 5 Department of Epidemiology, “Victor Babeș” University of Medicine and Pharmacy, Eftimie Murgu Square 2, 300041 Timișoara, Romania; 6 Doctoral School, Department of General Medicine, “Victor Babeș” University of Medicine and Pharmacy, Eftimie Murgu Square 2, 300041 Timișoara, Romania; emilia.clej@umft.ro

**Keywords:** insulin resistance, HOMA-IR, tumor necrosis factor-alpha, uric acid, leptin, adiponectin, type 2 diabetes, cardiovascular risk

## Abstract

*Background and Objectives*: Anthropometric measures incompletely capture the inflammatory and adipokine heterogeneity underlying insulin resistance. We examined associations of tumor necrosis factor-alpha (TNF-α), serum uric acid, and the leptin/adiponectin ratio with HOMA-IR after adjustment. *Materials and Methods*: This single-center cross-sectional secondary analysis included 147 of 239 hospitalized adults with HOMA-IR and predefined core data; fasting glucose, insulin, and biomarker testing was elective and patient-funded. Continuous ln(HOMA-IR) was the primary outcome. A secondary upper-quartile phenotype (HOMA-IR ≥ 4.78; *n* = 37) was cohort-specific and non-diagnostic. Robust linear regression used HC3 standard errors. Sensitivity analyses excluded T2DM and replaced BMI with waist circumference or visceral fat area. Binary and AUC analyses were exploratory (expanded complete-case model, *n* = 112). *Results*: Each doubling of (TNF-α + 1) was associated with a 10.2% higher HOMA-IR in the expanded model (*p* = 0.021). The association persisted after excluding T2DM (12.1% higher HOMA-IR per doubling; *n* = 81; *p* = 0.012) and when BMI was replaced by waist circumference (*p* = 0.022) or visceral fat area (*p* = 0.018). Uric acid and the leptin/adiponectin ratio did not reach statistical significance in the adjusted continuous-outcome model. The 112 complete cases and 35 incomplete cases were broadly similar in measured baseline characteristics, although uric acid was higher among complete cases (*p* = 0.020). *Conclusions*: In this selected hospitalized cohort, TNF-α showed the most consistent adjusted association with the observed fasting glucose-insulin phenotype. The binary and discrimination analyses remain preliminary and do not establish diagnostic or clinical prediction utility. Prospective external validation is required.

## 1. Introduction

Insulin resistance is a central pathophysiological feature of type 2 diabetes mellitus (T2DM) and an important component of cardiovascular–kidney–metabolic disease. It promotes dysglycemia, atherogenic dyslipidemia, endothelial dysfunction, hypertension, and prothrombotic and inflammatory signaling, while also identifying patients at increased long-term cardiovascular risk [1,2,3,4,5,6]. Contemporary guidance therefore emphasizes integrated assessment of glycemia, adiposity, blood pressure, lipids, renal status, and established cardiovascular disease rather than glucose control in isolation [1,2,3,4,5].

Anthropometric measures are clinically useful but imperfect surrogates of metabolic dysfunction. Individuals with similar body mass index (BMI), waist circumference, or measured visceral fat may have substantially different insulin sensitivity, adipokine profiles, inflammatory activity, and cardiometabolic trajectories [5,7,8,9]. This heterogeneity is particularly relevant in hospitalized internal medicine populations, in whom obesity and hypertension are common and may reduce the discriminatory value of conventional risk factors.

Tumor necrosis factor-α (TNF-α) is a biologically plausible mediator of obesity-associated insulin resistance. Experimental work demonstrated increased adipose TNF-α expression in obesity and improvement in insulin signaling after disruption of TNF pathways [10,11,12]. Human experimental and observational studies have also linked TNF-α signaling with impaired glucose disposal and future diabetes risk, although effect sizes vary according to population, assay, and covariate adjustment [13,14,15,16].

Adiponectin and leptin provide complementary information about adipose-tissue function. Adiponectin is generally insulin-sensitizing, whereas leptin concentrations rise with adiposity and may reflect leptin resistance. Their ratio can therefore integrate opposing adipokine signals and has correlated with insulin resistance in several adult cohorts [17,18,19,20,21]. Serum uric acid is another readily available metabolic marker. Hyperuricemia frequently clusters with insulin resistance, hypertension, and cardiovascular risk, but bidirectionality and residual confounding remain important concerns [22,23,24,25,26].

The present study addressed a research question distinct from our companion analysis of subclinical carotid remodeling in the same source cohort. We hypothesized that TNF-α, serum uric acid, and leptin/adiponectin imbalance would be associated with higher HOMA-IR after accounting for a parsimonious set of clinical covariates. The primary objective was to quantify associations with continuous HOMA-IR. The secondary, explicitly exploratory objective was to examine whether a biomarker-enriched model showed higher discrimination of a cohort-specific upper-quartile HOMA-IR phenotype than a parsimonious clinical comparator; this analysis was not intended to develop or validate a clinical prediction tool.

## 2. Materials and Methods

### 2.1. Study Design and Setting

We conducted a single-center observational cross-sectional secondary analysis of adults admitted to the Internal Medicine II Department of Clinical CF Hospital Timișoara, Romania, between 1 July 2025 and 30 June 2026. The manuscript was prepared in accordance with the Strengthening the Reporting of Observational Studies in Epidemiology (STROBE) recommendations [27].

The same source cohort has also been used in companion work focused on subclinical carotid remodeling, which addresses a separate research question and is reported separately. The present manuscript addresses a different research question, with HOMA-IR as the primary outcome, a distinct inflammatory–metabolic biomarker hypothesis, and a separate statistical framework. No carotid outcome or carotid-derived variable is analyzed in the present report. Cohort reuse is disclosed explicitly, here and in the accompanying cover letter, to permit assessment of participant overlap and to avoid duplicate interpretation of the same research question.

### 2.2. Study Population and Cohort Construction

The source database comprised 239 patient-level admission records. Eligibility for the present secondary analysis required age ≥ 18 years, one analyzable admission record, HOMA-IR, and the predefined minimum clinical and anthropometric dataset (age, sex, documented diabetes status, hypertension status, systolic and diastolic blood pressure, total cholesterol, triglycerides, HDL cholesterol, BMI, and visceral fat area). HOMA-IR required fasting glucose and fasting insulin measurements. These supplementary measurements were not universally available through the hospital laboratory: patients could elect to have them performed privately at Bioclinica Laboratory, Timisoara, at their own expense, and not all patients chose to undergo the additional testing. Consequently, patients without the measurements required to calculate HOMA-IR could not contribute to the primary HOMA-IR analysis. After application of the predefined core-data criteria, 89 of 239 records (37.2%) had at least one required clinical or anthropometric core variable unavailable, leaving 150 eligible records. A further 3 eligible patients had no HOMA-IR because they did not undergo the elective, self-funded fasting glucose/insulin testing. In total, 92 of 239 records (38.5%) could not enter the analytic cohort, leaving 147 patients. Biomarker availability was not required for entry into the 147-patient cohort and therefore varied by analysis. No missing core or biomarker values were imputed; regression analyses used complete cases for the variables included in each model. Because access to privately obtained supplementary testing depended on patient choice and willingness to self-fund testing, selection into biomarker-based complete-case analyses may not have been random. A comprehensive comparison of the 147 included patients with the 92 excluded source records was not possible because missing core variables were themselves the principal reason for exclusion. A post hoc comparison between the 112 biomarker-complete and 35 biomarker-incomplete participants was performed using available baseline variables; similarity on measured characteristics does not establish random biomarker availability. This unresolved selection mechanism is treated as a major limitation, and all inferences are restricted to the analyzed participants. No patient identifiers were included in the analytic dataset.

### 2.3. Clinical, Anthropometric, and Laboratory Variables

The dataset included age, sex, documented type 2 diabetes mellitus (T2DM), hypertension, smoking status, systolic and diastolic blood pressure, fasting glucose, fasting insulin, total cholesterol, triglycerides, high-density lipoprotein cholesterol (HDL-C), low-density lipoprotein cholesterol (LDL-C), gamma-glutamyl transferase (GGT), serum uric acid, body weight, height, body mass index (BMI), waist and hip circumferences, body-fat percentage, visceral fat area (VFA), adiponectin, leptin, tumor necrosis factor-α (TNF-α), and interleukin-6 (IL-6). BMI was calculated as body weight in kilograms divided by height in meters squared (kg/m^2^).

All venous blood samples were collected at Clinical CF Hospital Timișoara.

Routine biochemical measurements, including total cholesterol, triglycerides, HDL-C, LDL-C, GGT, and serum uric acid, were performed in the hospital clinical laboratory using validated automated clinical chemistry methods in routine clinical use.

Fasting plasma glucose and fasting serum insulin were measured at Bioclinica Laboratory, Timișoara. According to the laboratory’s routine assay documentation, glucose was measured by photometric/spectrophotometric methods and insulin by chemiluminescence/electrochemiluminescence.

Serum adiponectin and leptin were measured by enzyme immunoassay (EIA), TNF-α by chemiluminescence, and IL-6 by electrochemiluminescence at Bioclinica Laboratory, according to the laboratory procedures in routine use at the time of testing. This study is a secondary analysis of results already recorded in the analytical database. Manufacturer-specific reagent or platform identifiers, analytical measuring ranges, lower limits of detection, and intra- and inter-assay coefficients of variation were not retained in that database and could not be reconstructed reliably; therefore, these details are not inferred retrospectively. This limitation is relevant to reproducibility, and absolute biomarker concentrations in the present study should not be interpreted as assay-independent diagnostic or therapeutic cutoffs.

The supplementary fasting glucose, insulin, and biomarker investigations performed at Bioclinica were paid directly by the patients who elected to undergo them; they were not financed by the study. Accordingly, availability of these measurements reflects an elective, self-funded testing process rather than universal measurement across all admissions.

Anthropometric measurements were obtained during hospitalization using standardized procedures. Body composition, including body-fat percentage and visceral fat area, was assessed using the InBody 770 body composition analyzer (InBody Co., Ltd., Seoul, Republic of Korea). The device uses direct segmental multi-frequency bioelectrical impedance analysis (DSM-BIA) with an eight-point tactile electrode system and provides visceral fat area directly in cm^2^. Measurements were performed with participants barefoot and in light clothing, after removal of metallic accessories, following bladder emptying and before food intake, with participants maintaining an upright position before and throughout the examination. During measurement, the feet were positioned on the foot electrodes and the participants held the hand electrodes with their arms extended away from the trunk, according to the manufacturer’s standardized testing procedure. Visceral fat area and percentage body fat data generated by the device were recorded directly in the study database.

### 2.4. Biomarker Measurements

TNF-α and IL-6 concentrations were expressed in pg/mL, leptin concentrations in ng/mL, and adiponectin concentrations in µg/mL. The leptin/adiponectin ratio was calculated as leptin (ng/mL) divided by adiponectin (µg/mL) when both biomarkers were available and adiponectin was greater than zero. TNF-α had a right-skewed distribution and included values close to zero. It was therefore modeled as log_2_(TNF-α + 1): adding 1 preserved zero-valued observations, the logarithmic transformation reduced right skew and the leverage of extreme concentrations, and base 2 permitted interpretation of the coefficient for each doubling of (TNF-α + 1). The leptin/adiponectin ratio was also right-skewed and was modeled on the log_2_ scale; all observed ratios were positive.

### 2.5. Outcomes

The primary outcome was HOMA-IR, calculated from fasting measurements using the conventional formula: fasting insulin (µU/mL) × fasting plasma glucose (mmol/L)/22.5 [28,29]. Glucose values recorded in mg/dL were converted to mmol/L by dividing by 18.0 before HOMA-IR calculation. HOMA-IR was natural-log transformed in regression models because of right-skew. For secondary analyses, marked insulin resistance was defined as the upper quartile of the cohort distribution (HOMA-IR ≥ 4.78). This threshold was explicitly exploratory and cohort-specific and should not be interpreted as a universal diagnostic cutoff. Because HOMA-IR is a fasting glucose–insulin surrogate, its interpretation in established T2DM may be influenced by β-cell reserve and glucose-lowering treatment, including possible exogenous insulin exposure. T2DM status was therefore included a priori in multivariable models. Diabetes duration, medication class, dose, and treatment timing relative to sampling were unavailable, so the primary estimand should be interpreted as an association with the observed fasting glucose–insulin phenotype in this mixed hospitalized cohort rather than as direct quantification of tissue insulin sensitivity in treated diabetes. The study was not designed to establish a diagnostic HOMA-IR threshold.

### 2.6. Statistical Analysis

Continuous variables were summarized as mean ± standard deviation when approximately symmetric and as median [interquartile range] when skewed. Categorical variables were expressed as counts and percentages. Marked versus lower insulin-resistance groups were compared with the Mann–Whitney U test for continuous variables and Fisher’s exact test for categorical variables. Spearman coefficients quantified unadjusted correlations with HOMA-IR. All tests were two-sided, and *p* < 0.05 was considered statistically significant. Secondary comparisons were exploratory and were not adjusted for multiplicity.

The prespecified parsimonious association model used ln(HOMA-IR) as the dependent variable and included log_2_(TNF-α + 1), age, sex, documented T2DM, and BMI. An expanded model additionally included log_2_(leptin/adiponectin ratio) and serum uric acid. The clinical covariate set was intentionally restricted to age, sex, T2DM, and BMI to limit model complexity. Heteroscedasticity-consistent HC3 standard errors were used. Exponentiated continuous-model coefficients are presented as percentage differences in HOMA-IR. Sensitivity analyses of the expanded continuous model (i) excluded patients with documented T2DM and omitted T2DM from the covariate set and (ii) replaced BMI, one at a time, with waist circumference or visceral fat area while retaining all other covariates. A further sensitivity analysis excluded TNF-α values above the 99th percentile.

For the secondary binary outcome, logistic regression compared the parsimonious clinical comparator (age, sex, T2DM, and BMI) with an expanded model additionally containing TNF-α, leptin/adiponectin ratio, and uric acid. Both models were fitted on the same complete-case sample (*n* = 112) so that they remained nested and directly comparable. Apparent discrimination was quantified using the AUC. Percentile confidence intervals for each AUC and their difference were obtained from 3000 nonparametric bootstrap samples. Internal validation used stratified five-fold cross-validation with a fixed random seed. No missing values were imputed. Continuous HOMA-IR remained the primary outcome. The upper-quartile threshold was internally derived, discards information, and is not a clinical definition. With no more than 30 upper-quartile observations among 112 complete cases and seven covariates in the expanded model, the event-to-parameter ratio was fewer than five and coefficient and discrimination estimates were vulnerable to overfitting. Cross-validation was used only as a limited internal robustness check and cannot correct inadequate effective sample size. No clinical score, probability threshold, or decision rule was derived, and the analysis was not prediction-model development. Descriptive statistics, between-group comparisons, and Spearman correlations were performed using IBM SPSS Statistics for Windows, version 26.0 (IBM Corp., Armonk, NY, USA). Regression modelling with HC3 heteroscedasticity-consistent standard errors, nonparametric bootstrap estimation of AUC confidence intervals, stratified five-fold cross-validation, and figure generation were performed in Python version 3.12 using statsmodels, scikit-learn, NumPy, and Matplotlib version 3.9.2 (Matplotlib Development Team).

Because this was a secondary analysis of an existing dataset, no a priori sample-size calculation was performed; the available analytic sample determined precision. To evaluate complete-case selection, baseline clinical, anthropometric, and routinely available laboratory characteristics were compared between the 112 participants included in the expanded model and the 35 participants excluded because at least one required biomarker was unavailable. Welch’s *t* test was used for approximately symmetric continuous variables, the Mann–Whitney U test for skewed continuous variables, and Fisher’s exact test for categorical variables. These post hoc comparisons were descriptive and were not adjusted for multiplicity.

### 2.7. Ethics

This secondary analysis was conducted in accordance with the Declaration of Helsinki and was approved by the Ethics Council of Clinical CF Hospital Timișoara (approval no. 5958/30 July 2026). The approval covered secondary use of data from patients admitted to the Internal Medicine II Department between 1 November 2024 and 30 June 2026, whereas the present analysis was restricted to admissions from 1 July 2025 through 30 June 2026. At hospital admission, all patients had provided written informed consent, including authorization for the anonymized use of clinical data for scientific research. The supplementary privately funded laboratory measurements included in this secondary dataset were available only for patients who had elected to undergo them. No additional patient contact, intervention, or new sample collection was undertaken for the purpose of the present secondary analysis, and the analytical dataset was fully de-identified before statistical processing.

## 3. Results

### 3.1. Cohort Characteristics and Biomarker Availability

Of 239 source records, 89 (37.2%) had at least one required clinical or anthropometric core variable unavailable, leaving 150 eligible records; a further 3 patients had no HOMA-IR because the elective, self-funded fasting glucose/insulin testing was not undertaken. The remaining 147 patients constituted the primary analytic cohort. Their mean age was 55.0 ± 10.1 years, and 76 (51.7%) were men. T2DM was documented in 39 (26.5%), hypertension in 125 (85.0%), and current smoking in 28 (19.0%). Mean BMI was 34.1 ± 4.9 kg/m^2^. TNF-α was available in 121 patients, adiponectin in 122, leptin in 117, the leptin/adiponectin ratio in 115, serum uric acid in 144, and IL-6 in 117. The complete expanded-model population comprised 112 patients. Participant selection and biomarker availability are summarized in Figure 1 and Table 1.

The 112 complete cases and 35 participants excluded from the expanded model because of incomplete biomarker data were similar with respect to age, sex, T2DM, hypertension, smoking, HOMA-IR, fasting glucose and insulin, BMI, waist circumference, visceral fat area, and GGT (all *p* > 0.05). Serum uric acid was higher among complete cases (median 5.95 vs. 5.50 mg/dL; *p* = 0.020). These comparisons are descriptive and do not establish that biomarker availability was random (Table 2).

### 3.2. Phenotype of Cohort-Defined Marked Insulin Resistance

The cohort-specific upper-quartile HOMA-IR phenotype was present in 37 patients (25.2%). Compared with participants below the upper quartile, these patients more frequently had T2DM (16/37 vs. 23/110; *p* = 0.010) and had higher fasting glucose and insulin. In contrast, BMI, waist circumference, visceral fat area, triglycerides, and HDL cholesterol did not differ materially between groups. These unadjusted comparisons are descriptive and do not establish a clinically distinct or diagnostically validated phenotype. Detailed comparisons are presented in Table 3.

The cohort-defined marked-insulin-resistance group had higher TNF-α, serum uric acid, leptin, leptin/adiponectin ratio, and GGT. The distribution of TNF-α according to cohort-specific insulin-resistance status is illustrated in Figure 2, showing the separation in TNF-α values between the two groups. IL-6 and adiponectin alone did not differ significantly. This pattern indicates inflammatory–metabolic heterogeneity that was not captured by anthropometry in this cohort.

### 3.3. Unadjusted Correlations with HOMA-IR

The leptin/adiponectin ratio showed the strongest unadjusted biomarker correlation with HOMA-IR (Spearman ρ = 0.282; *p* = 0.002). Adiponectin was inversely correlated with HOMA-IR (ρ = −0.228; *p* = 0.011), whereas TNF-α (ρ = 0.191; *p* = 0.036) and uric acid (ρ = 0.168; *p* = 0.045) showed modest positive correlations. IL-6, BMI, waist circumference, and visceral fat area were not significantly correlated with HOMA-IR. The complete set of unadjusted Spearman correlations with HOMA-IR is summarized in Table 4.

### 3.4. Multivariable Associations with Continuous HOMA-IR

In the parsimonious model (*n* = 121), each doubling of TNF-α + 1 was associated with an 8.9% higher HOMA-IR (β = 0.085; 95% CI 0.000 to 0.169, the lower limit lying above zero before rounding; *p* = 0.049). Documented T2DM was associated with a 41.6% higher HOMA-IR (*p* = 0.023), whereas age, sex, and BMI were not independently associated. The adjusted R^2^ was 0.057. After excluding TNF-α values above the 99th percentile, the association remained significant (β = 0.100; 95% CI 0.002–0.197; *p* = 0.045; *n* = 119).

In the expanded complete-case model (*n* = 112), each doubling of (TNF-α + 1) was associated with a 10.2% higher HOMA-IR (β = 0.097; 95% CI 0.015 to 0.178; *p* = 0.021). The leptin/adiponectin ratio (*p* = 0.064) and uric acid (*p* = 0.096) did not reach the conventional *p* < 0.05 threshold after multivariable adjustment, while T2DM remained significant (*p* = 0.012). The adjusted R^2^ was 0.129. Complete estimates are presented in Table 5.

After exclusion of participants with documented T2DM, the expanded continuous-outcome model included 81 complete cases. The TNF-α association remained significant: each doubling of (TNF-α + 1) corresponded to a 12.1% higher HOMA-IR (β = 0.114; 95% CI 0.025 to 0.204; *p* = 0.012). The leptin/adiponectin ratio (β = 0.060; *p* = 0.288), uric acid (β = 0.022; *p* = 0.537), age, sex, and BMI were not statistically significant; adjusted R^2^ was 0.052. In the full 112-person complete-case sample, replacing BMI with waist circumference yielded a similar TNF-α estimate (β = 0.096; 95% CI 0.014 to 0.178; *p* = 0.022), while waist circumference was not associated with HOMA-IR (*p* = 0.971). Replacing BMI with visceral fat area likewise preserved the TNF-α association (β = 0.096; 95% CI 0.016 to 0.176; *p* = 0.018), while visceral fat area was not associated with HOMA-IR (*p* = 0.915). These sensitivity analyses are summarized in Table 5.

### 3.5. Associations with the Cohort-Defined Marked Insulin-Resistance Phenotype

In the exploratory expanded logistic association model (*n* = 112), TNF-α was associated with the cohort-defined marked-insulin-resistance phenotype after multivariable adjustment (OR 1.64 per two-fold increase in [TNF-α + 1]; 95% CI 1.14–2.35; *p* = 0.007), as was serum uric acid (OR 1.65 per 1 mg/dL; 95% CI 1.16–2.35; *p* = 0.005). T2DM remained the strongest clinical correlate (OR 6.30; 95% CI 2.01–19.77; *p* = 0.002). The leptin/adiponectin ratio did not reach conventional statistical significance after adjustment (OR 1.41 per doubling; *p* = 0.066); age, sex, and BMI were also not significant. Adjusted effect estimates for all covariates are reported in Table 6 and visually summarized in Figure 3. These estimates describe associations with a cohort-defined phenotype and are not validated predictive effects.

The parsimonious clinical comparator had an apparent AUC of 0.690 (bootstrap 95% CI 0.561–0.804), whereas the biomarker-enriched model had an apparent AUC of 0.815 (95% CI 0.711–0.908), corresponding to an absolute apparent difference of 0.125 (bootstrap 95% CI 0.025–0.233). Model fit differed by likelihood-ratio testing (*p* < 0.001). Pooled five-fold cross-validated AUCs were 0.611 for the parsimonious comparator and 0.750 for the expanded model. These values are reported as exploratory discrimination estimates only. The limited sample, complete-case restriction, and low number of marked-insulin-resistance observations relative to model complexity preclude claims of clinical prediction utility or transportability. Apparent ROC curves are shown in Figure 4.

## 4. Discussion

In this selected hospital cohort, TNF-α showed the most consistent association with HOMA-IR. The finding persisted after exclusion of participants with documented T2DM and after replacing BMI with waist circumference or visceral fat area, whereas the alternative anthropometric indices were not independently associated with HOMA-IR. Serum uric acid was associated with the cohort-specific upper-quartile phenotype but not with continuous HOMA-IR after adjustment. The leptin/adiponectin ratio showed the strongest unadjusted biomarker correlation, but its adjusted estimates remained uncertain. The biomarker-enriched model showed higher apparent and cross-validated AUCs than the deliberately parsimonious comparator; given the effective sample size, this remains a preliminary signal rather than evidence of clinical prediction utility.

The strong association between documented T2DM and the high-HOMA phenotype requires careful interpretation. HOMA-IR is an epidemiological surrogate derived from fasting glucose and insulin and is not equivalent to clamp-measured peripheral insulin sensitivity [28,29]. In established diabetes, β-cell dysfunction and glucose-lowering therapy can alter fasting glucose and insulin concentrations independently of tissue insulin sensitivity. Because diabetes duration, medication class, treatment intensity, timing of therapy relative to sampling, and exogenous insulin use were not available in the analytical dataset, the present results should be interpreted as associations with the observed fasting glucose–insulin phenotype rather than as definitive quantification of tissue insulin resistance in treated T2DM. Inclusion of documented T2DM as an a priori covariate reduces, but cannot eliminate, this source of heterogeneity. Replication in a prospectively characterized non-diabetic or treatment-stratified cohort would therefore be especially informative.

The TNF-α findings are biologically coherent. Seminal experimental studies demonstrated increased adipose TNF-α expression in obesity and a direct effect on insulin signaling [10,11,12]. In humans, TNF-α infusion can impair insulin-stimulated glucose uptake, and circulating inflammatory markers have predicted future diabetes in population studies [13,14,15,16]. TNF-α can activate stress kinases, promote inhibitory serine phosphorylation of insulin-receptor substrates, alter adipocyte lipolysis, and interact with endothelial and hepatic pathways. The modest variance explained in our models is also plausible: insulin resistance is multifactorial, and a single circulating cytokine cannot capture its full tissue-specific biology.

The lack of association between IL-6 and HOMA-IR should not be interpreted as evidence against inflammatory involvement. IL-6 has context-dependent metabolic effects, differs between acute and chronic exposure, and is sensitive to assay detection, sampling conditions, adipose distribution, exercise, intercurrent illness, and medication use. Both the median and the lower quartile of IL-6 were 0.00 pg/mL, so at least half of the participants with an IL-6 measurement had a zero-coded value; these values may reflect concentrations below the laboratory reporting threshold or database coding and therefore limit mechanistic interpretation of the null association.

The adipokine results support the concept that functional adipose-tissue phenotype may be more informative than adiposity alone. Lower adiponectin and higher leptin tend to accompany insulin resistance, and ratios combining these opposing signals have shown stronger associations than either biomarker individually in several cohorts [17,18,19,20,21]. In our data, the leptin/adiponectin ratio was approximately twice as high in the marked-insulin-resistance group and correlated with HOMA-IR, whereas BMI and visceral fat were nearly identical between groups. This discordance is clinically interesting because it suggests that patients with comparable body size can differ materially in endocrine adipose function.

Serum uric acid also showed clinically plausible metabolic information, particularly in the exploratory binary analysis. Uric acid may be both a marker and mediator of insulin resistance through oxidative stress, endothelial dysfunction, altered nitric-oxide signaling, mitochondrial effects, and adipose inflammation [22,23,24,25,26]. Conversely, hyperinsulinemia can reduce renal urate excretion, creating a bidirectional relationship. In the present data, uric acid was associated with the cohort-defined marked-insulin-resistance phenotype after adjustment, whereas its association with continuous HOMA-IR in the expanded model did not reach the conventional significance threshold. The cross-sectional design cannot determine directionality, and renal function, diuretic use, diet, alcohol exposure, and purine metabolism were not comprehensively modeled. The finding is therefore best regarded as hypothesis-generating rather than evidence that uric acid itself improves clinical detection of insulin resistance. In particular, the absence of renal-function and diuretic-treatment data prevents separation of metabolic from renal or treatment-related determinants of serum urate.

A notable feature was the absence of meaningful group differences in BMI, waist circumference, and visceral fat area. The cohort had a high burden of obesity and hypertension, which may have restricted variability and attenuated anthropometric associations. It is also possible that a single body-composition measurement does not fully reflect ectopic hepatic or intramuscular fat, adipocyte hypertrophy, fibrosis, immune-cell infiltration, or adipokine secretion [7,8,9]. These findings align with the cardiovascular–kidney–metabolic framework, in which excess adiposity is one component of a broader pathobiological continuum rather than a complete description of metabolic risk [3,4,5].

The observed difference in AUC after adding inflammatory and metabolic biomarkers to a deliberately parsimonious comparator is preliminary and should not be interpreted as incremental clinical value. The expanded model contained seven covariates but no more than 30 upper-quartile observations among 112 complete cases, yielding fewer than five events per variable and a substantial risk of overfitting. The comparator was not an optimized clinical risk model; a more comprehensive comparator could perform differently. Five-fold cross-validation attenuated the apparent AUCs but cannot compensate for limited effective sample size or substitute for penalized model development and independent external validation. Accordingly, the AUC analysis is retained only as a hypothesis-generating secondary analysis. A future study should prespecify biomarker handling and a clinically justified comparator, recruit a substantially larger longitudinal cohort, measure HbA1c, renal function, C-reactive protein, medication intensity, diabetes duration, and cardiovascular outcomes, and use penalization or shrinkage together with external validation, calibration assessment, and decision-curve analysis.

Clinically, the results do not justify routine TNF-α or adipokine testing. Instead, they support a research strategy that distinguishes anatomical adiposity from metabolic inflammation and endocrine adipose dysfunction. For an internal medicine population with prevalent obesity, hypertension, and diabetes, this distinction may help explain why conventional anthropometry alone incompletely captures dysmetabolic severity.

### Strengths and Limitations

This study has several strengths that support the internal consistency of its findings while remaining compatible with an exploratory interpretation. The analysis integrated clinical, anthropometric, biochemical, inflammatory, and adipokine data within the same hospital cohort. HOMA-IR was evaluated primarily as a continuous outcome and secondarily as a cohort-specific categorical phenotype. The statistical approach included heteroscedasticity-robust standard errors, exclusion of extreme TNF-α values, exclusion of participants with T2DM, alternative adjustment for waist circumference and visceral fat area, a complete-case comparison, bootstrap estimation of AUC uncertainty, and stratified five-fold cross-validation. The distinction between association analysis and prediction-model development is explicit, and reuse of the source cohort for a separate carotid-remodeling analysis is disclosed.

Several limitations nevertheless define the boundaries within which the results should be interpreted. First, the cross-sectional design precludes determination of temporality or causality. The observed relationships between TNF-α, uric acid, adipokine imbalance, and HOMA-IR are associations and do not demonstrate that these biomarkers cause insulin resistance. HOMA-IR is an established epidemiological surrogate rather than a direct measurement of peripheral insulin sensitivity by hyperinsulinemic–euglycemic clamp. Interpretation is additionally complex in established T2DM because diabetes duration, β-cell reserve, glucose-lowering medication class and dose, and possible exogenous insulin use were not available. Accordingly, results in patients with T2DM should not be interpreted as assay-independent quantification of tissue insulin resistance.

Second, selection and missing-data mechanisms may affect both internal and external validity. Of 239 source records, 89 (37.2%) lacked at least one required clinical or anthropometric core variable, and the final analytic cohort comprised 147 participants; the biomarker-enriched complete-case model included 112. Supplementary testing was elective and self-funded, creating a plausible non-random selection mechanism related to willingness or ability to pay. In the post hoc comparison, complete and incomplete cases were broadly similar across measured baseline characteristics, but serum uric acid was higher among complete cases. This isolated difference and the absence of socioeconomic, treatment, and acute-illness descriptors mean that a missing-at-random mechanism cannot be established. No values were imputed, and generalizability remains limited to the analyzed participants.

Third, residual and unmeasured confounding remains possible. The available database did not permit comprehensive adjustment for several variables that may influence insulin sensitivity, inflammatory activity, or urate metabolism, including HbA1c, diabetes duration and treatment intensity, renal function, albuminuria, C-reactive protein, acute infection or inflammatory status, dietary and alcohol exposure, physical activity, urate-lowering therapy, diuretic exposure, medication doses, and detailed cardiovascular history. This issue is particularly relevant in a hospitalized internal-medicine population, because acute illness may influence circulating cytokines independently of chronic metabolic inflammation. For serum uric acid specifically, the absence of renal-function and diuretic-treatment data is an important source of residual confounding. Manufacturer-specific assay platforms, analytical ranges, lower limits of detection, and intra- and inter-assay coefficients of variation were not retained in the secondary analytical dataset; therefore, absolute biomarker values should not be transported across assays or used as clinical cutoffs. IL-6 was recorded as zero in at least half of the participants with a measurement (median and lower quartile both 0.00 pg/mL); these values may reflect concentrations below the laboratory reporting threshold rather than true absence of circulating IL-6, and they limit mechanistic interpretation of the null IL-6 finding.

Finally, several secondary comparisons were exploratory and were not adjusted for multiplicity. The continuous HOMA-IR model is therefore the principal inferential analysis. The binary analysis is especially vulnerable to model instability: only 37 patients met the cohort-defined marked-insulin-resistance criterion in the full cohort, while the expanded complete-case model (*n* = 112) contained seven covariates and no more than 30 events, corresponding to fewer than five events per variable. Apparent AUC improvement and adjusted odds ratios may therefore be optimistic despite bootstrap uncertainty estimates and five-fold cross-validation. The clinical comparator was deliberately parsimonious and does not exhaust routinely available metabolic risk information; consequently, the AUC difference should not be interpreted as proof that biomarker testing adds value over an optimized clinical model. The high prevalence of obesity and hypertension may also have restricted variability in anthropometric measures and limits generalizability to community-based, metabolically healthier, younger, or geographically diverse populations. Larger prospective studies using standardized assays, prespecified and adequately powered models, penalization or shrinkage where appropriate, and independent external validation are required before clinical implementation can be considered.

## 5. Conclusions

In this selected sample of 147 hospitalized adults, TNF-α showed the most consistent adjusted association with the observed fasting glucose-insulin phenotype. This association persisted after excluding T2DM and after alternative adjustment for waist circumference or visceral fat area. Serum uric acid and the leptin/adiponectin ratio showed less consistent exploratory signals. The data do not establish causality, tissue-level insulin sensitivity, a diagnostic HOMA-IR threshold, or clinical prediction value. Non-random selection related to elective self-funded testing, incomplete biomarker panels, limited assay and treatment data, residual confounding, and modest effective sample size restrict inference. Prospective, adequately powered external validation remains necessary.

## Figures and Tables

**Figure 1:**
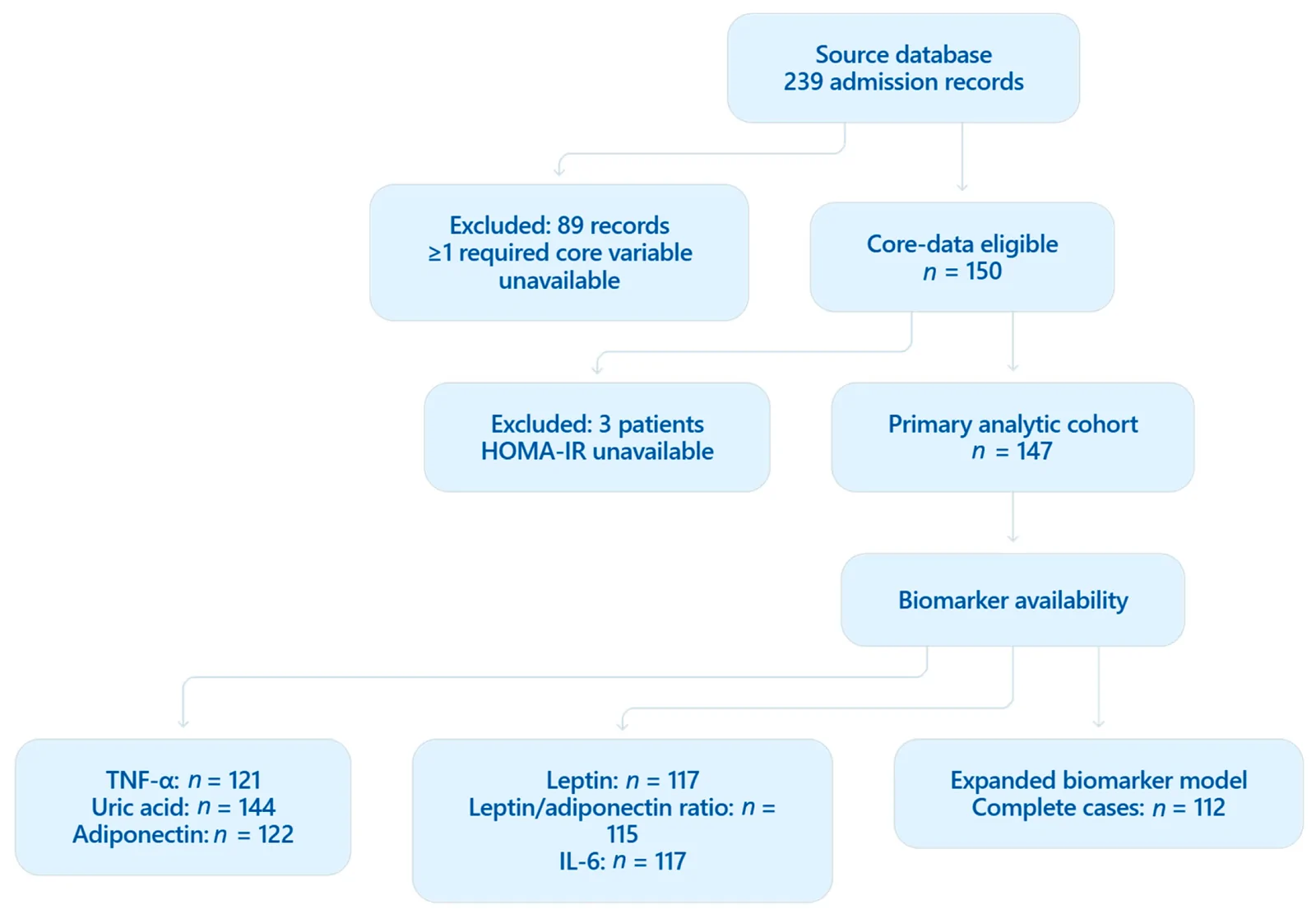
Participant flow and analysis-specific biomarker availability. The source database contained 239 admission records. The primary analytic cohort required HOMA-IR and predefined core clinical and anthropometric data. Biomarker analyses used available-case denominators, while the expanded biomarker model included 112 complete cases.

**Figure 2:**
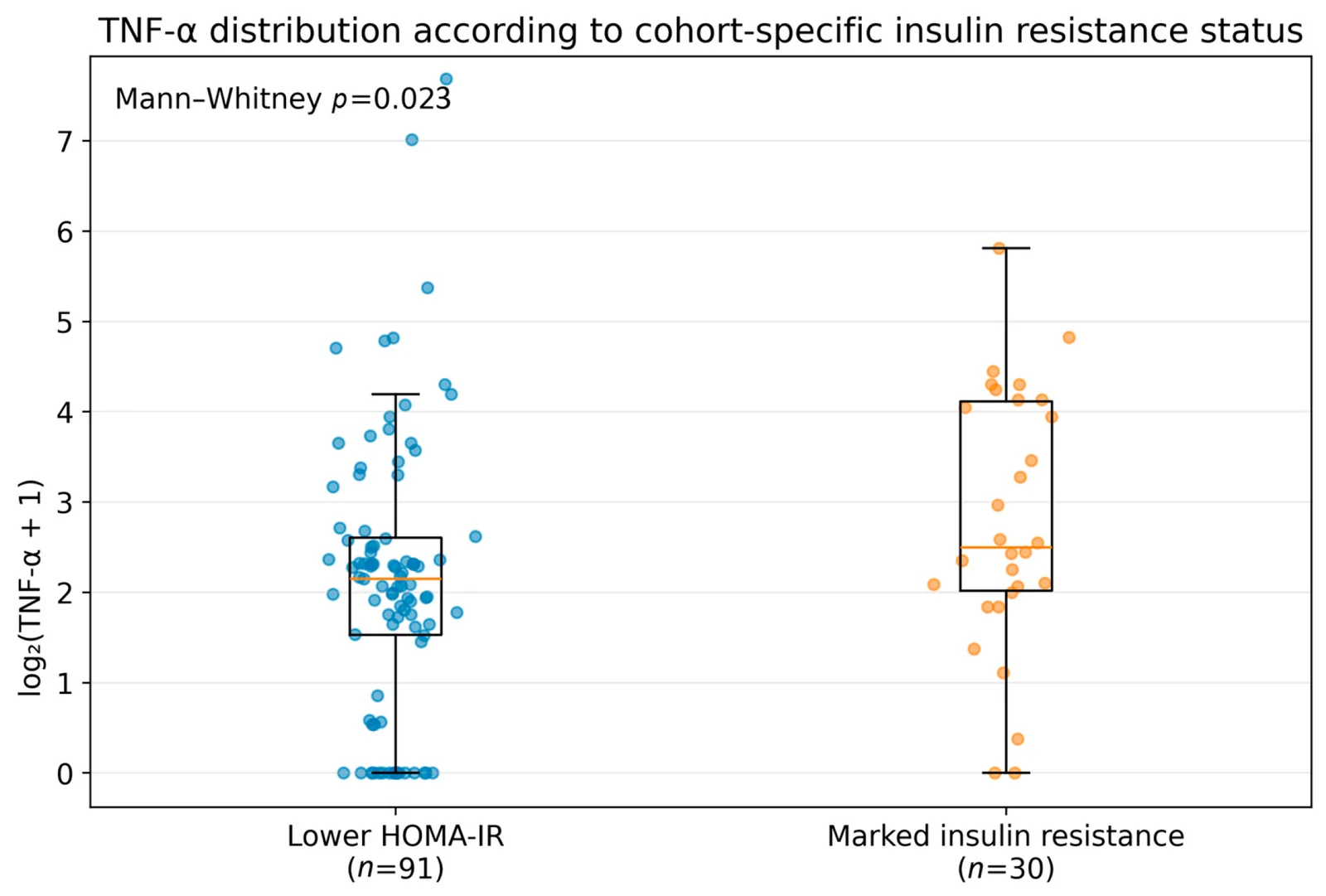
Distribution of log_2_(TNF-α + 1) among patients with lower versus marked insulin resistance. TNF-α concentrations were measured in pg/mL and were available for 121 participants; accordingly, the figure includes 91 patients in the lower-HOMA-IR group and 30 in the marked-insulin-resistance group. Individual observations are shown with box plots; the group comparison used raw TNF-α values and the Mann–Whitney U test. Blue circles represent participants in the lower HOMA-IR group, whereas orange circles represent participants with marked insulin resistance. Colors are used solely to distinguish the two study groups.

**Figure 3:**
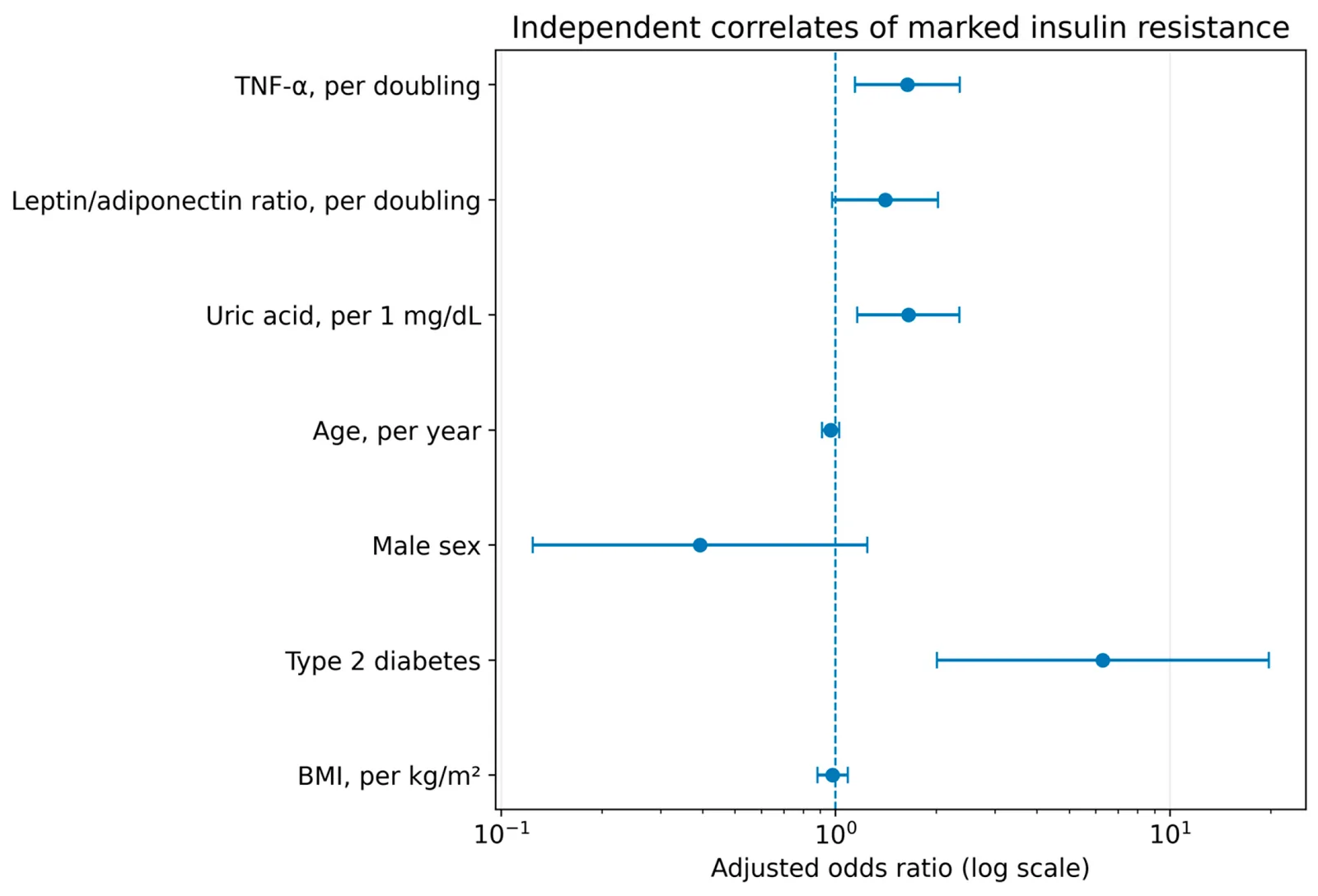
Adjusted odds ratios and 95% confidence intervals for the exploratory cohort-defined marked-insulin-resistance phenotype in the expanded association model. The x-axis is logarithmic. The plotted TNF-α effect corresponds to a one-unit increase in log_2_(TNF-α + 1), i.e., a doubling of (TNF-α + 1).

**Figure 4:**
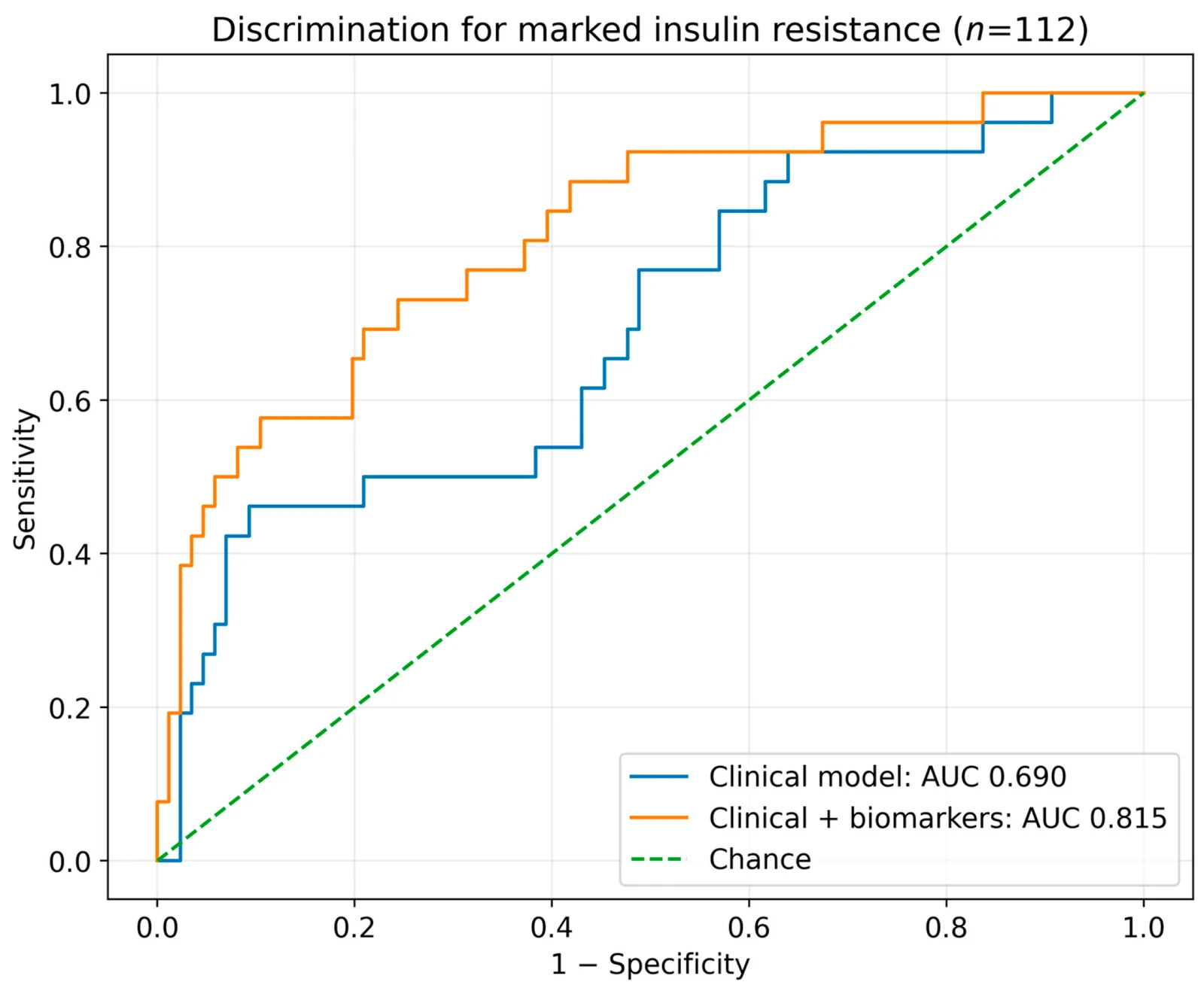
Receiver operating characteristic curves for the parsimonious clinical comparator (age, sex, T2DM, and BMI) and the biomarker-enriched model additionally including TNF-α, leptin/adiponectin ratio, and serum uric acid. Curves show apparent in-sample performance; pooled five-fold cross-validated AUCs are reported in the text. The figure is exploratory and does not represent external validation of a prediction model.

**Table 1:** Overall cohort characteristics and biomarker availability. Values are mean ± SD, median [IQR], or *n* (%).

Variable	Available *n*	Overall Value
Age, years	147	55.0 ± 10.1
Male sex	147	76 (51.7%)
Type 2 diabetes	147	39 (26.5%)
Hypertension	147	125 (85.0%)
Current smoking	147	28 (19.0%)
HOMA-IR	147	3.24 [2.20–4.78]
Fasting glucose, mg/dL	147	97.1 [86.5–115.0]
Fasting insulin, µU/mL	147	13.80 [9.25–18.40]
BMI, kg/m^2^	147	34.1 ± 4.9
Waist circumference, cm	147	109.0 [102.5–116.0]
Visceral fat area, cm^2^	147	173.5 [154.2–197.1]
GGT, U/L	147	33.70 [23.80–51.35]
Serum uric acid, mg/dL	144	5.90 [5.10–7.10]
TNF-α, pg/mL	121	3.77 [2.13–8.71]
Adiponectin, µg/mL	122	7.13 [4.73–11.04]
Leptin, ng/mL	117	39.30 [20.30–67.44]
Leptin/adiponectin ratio	115	5.44 [2.78–11.33]
IL-6, pg/mL	117	0.00 [0.00–14.67]

**Table 2:** Baseline characteristics of participants included in versus excluded from the expanded complete-case model. Values are mean ± SD, median [IQR], or *n*/*N* (%). *p* values are from Welch’s *t* test, Mann–Whitney U test, or Fisher’s exact test, as appropriate. Comparisons are post hoc and were not adjusted for multiplicity. Serum uric acid was available in 32 of the 35 incomplete cases; all other variables were available for all 147 participants.

Variable	Complete Cases (*n* = 112)	Incomplete Cases (*n* = 35)	*p*-Value
Age, years	55.1 ± 10.2	54.8 ± 9.9	0.852
Male sex	58/112 (51.8%)	18/35 (51.4%)	1.000
Type 2 diabetes	31/112 (27.7%)	8/35 (22.9%)	0.664
Hypertension	96/112 (85.7%)	29/35 (82.9%)	0.786
Current smoking	25/112 (22.3%)	3/35 (8.6%)	0.086
HOMA-IR	3.17 [2.11–4.50]	3.65 [2.90–5.07]	0.241
Fasting glucose, mg/dL	97.65 [86.97–116.15]	96.60 [83.60–113.00]	0.346
Fasting insulin, µU/mL	13.45 [9.09–18.20]	14.50 [11.02–21.85]	0.141
BMI, kg/m^2^	34.1 ± 5.1	34.0 ± 4.3	0.876
Waist circumference, cm	109.50 [102.75–118.00]	109.00 [102.50–115.00]	0.588
Visceral fat area, cm^2^	173.45 [155.20–196.43]	173.60 [137.70–198.00]	0.553
GGT, U/L	33.55 [23.27–51.70]	38.40 [25.35–49.60]	0.938
Serum uric acid, mg/dL	5.95 [5.30–7.40]	5.50 [4.80–6.25]	0.020

**Table 3:** Clinical and biomarker characteristics according to the cohort-defined marked-insulin-resistance phenotype (upper-quartile HOMA-IR ≥ 4.78). Values are median [IQR] or *n*/*N* (%). *p* values are from Mann–Whitney U or Fisher’s exact tests. Biomarker denominators vary because of missing measurements.

Variable	Lower HOMA-IR (*n* = 110)	Marked IR (*n* = 37)	*p*-Value
Male sex	59/110 (53.6%)	17/37 (45.9%)	0.451
Type 2 diabetes	23/110 (20.9%)	16/37 (43.2%)	0.010
Hypertension	92/110 (83.6%)	33/37 (89.2%)	0.595
Current smoking	22/110 (20.0%)	6/37 (16.2%)	0.809
Age, years	55.0 [49.2–61.8]	56.0 [51.0–60.0]	0.480
HOMA-IR	2.84 [1.89–3.63]	6.24 [5.27–9.33]	<0.001
Fasting glucose, mg/dL	93.0 [83.3–110.4]	113.6 [98.0–126.8]	<0.001
Fasting insulin, µU/mL	11.20 [8.63–14.95]	23.30 [18.86–31.00]	<0.001
BMI, kg/m^2^	33.3 [30.4–36.8]	33.3 [30.6–38.2]	0.841
Waist circumference, cm	109.0 [103.0–116.0]	110.0 [102.0–118.0]	0.849
Visceral fat area, cm^2^	174.2 [154.6–197.7]	165.2 [152.2–193.7]	0.827
Triglycerides, mg/dL	184.6 [135.2–240.0]	161.5 [129.8–212.4]	0.478
HDL-C, mg/dL	47.0 [40.2–55.5]	46.7 [38.5–57.0]	0.528
GGT, U/L	31.45 [23.12–46.83]	46.00 [28.30–57.50]	0.031
Serum uric acid, mg/dL	5.70 [4.97–6.80]	6.50 [5.50–7.60]	0.020
TNF-α, pg/mL	3.44 [1.89–5.10]	4.65 [3.04–16.32]	0.023
IL-6, pg/mL	0.00 [0.00–12.08]	0.00 [0.00–13.54]	0.931
Adiponectin, µg/mL	7.77 [4.76–11.25]	7.00 [4.11–9.00]	0.226
Leptin, ng/mL	34.60 [19.70–65.29]	55.85 [34.70–72.85]	0.045
Leptin/adiponectin ratio	4.65 [2.14–8.89]	8.88 [4.79–12.89]	0.015

**Table 4:** Spearman correlations between candidate clinical or biomarker variables and HOMA-IR.

Variable	*n*	Spearman *ρ*	*p*-Value
TNF-α	121	0.191	0.036
Visceral fat area	147	0.064	0.440
Serum uric acid	144	0.168	0.045
GGT	147	0.148	0.074
IL-6	117	−0.006	0.951
Adiponectin	122	−0.228	0.011
Leptin	117	0.181	0.051
Leptin/adiponectin ratio	115	0.282	0.002
BMI	147	0.034	0.679
Waist circumference	147	0.076	0.362

**Table 5:** Expanded heteroscedasticity-robust linear association models for ln(HOMA-IR). The primary expanded model included 112 complete cases. Sensitivity analyses excluded T2DM (*n* = 81) or replaced BMI with waist circumference or visceral fat area (*n* = 112). TNF-α was modeled as log_2_(TNF-α + 1), and the leptin/adiponectin ratio was log_2_-transformed. For these predictors, β corresponds to a one-unit increase on the log_2_ scale. Estimates are associative and should not be interpreted causally.

Predictor	β for ln(HOMA-IR)	95% CI	*p*-Value
TNF-α + 1, per doubling	0.097	0.015 to 0.178	0.021
Leptin/adiponectin ratio, per doubling	0.096	−0.005 to 0.197	0.064
Uric acid, per 1 mg/dL	0.054	−0.010 to 0.118	0.096
Age, per year	−0.003	−0.015 to 0.009	0.621
Male sex	0.041	−0.226 to 0.307	0.765
Type 2 diabetes	0.366	0.080 to 0.652	0.012
BMI, per kg/m^2^	−0.001	−0.025 to 0.023	0.930
Sensitivity analysis: participants without T2DM (*n* = 81)			
TNF-α + 1, per doubling	0.114	0.025 to 0.204	0.012
Leptin/adiponectin ratio, per doubling	0.060	−0.050 to 0.170	0.288
Uric acid, per 1 mg/dL	0.022	−0.048 to 0.092	0.537
Alternative anthropometric adjustment (*n* = 112)			
TNF-α + 1, per doubling; waist model	0.096	0.014 to 0.178	0.022
Waist circumference, per cm	0.0002	−0.012 to 0.012	0.971
TNF-α + 1, per doubling; visceral-fat model	0.096	0.016 to 0.176	0.018
Visceral fat area, per cm^2^	0.0002	−0.0032 to 0.0036	0.915

**Table 6:** Exploratory expanded logistic association model for the cohort-defined marked-insulin-resistance phenotype (upper-quartile HOMA-IR ≥ 4.78; *n* = 112). TNF-α was modeled as log_2_(TNF-α + 1), and the leptin/adiponectin ratio was log_2_-transformed; ORs correspond to a one-unit increase on the respective log_2_ scales. The model was not developed or validated as a clinical prediction tool.

Predictor	Adjusted OR	95% CI	*p*-Value
TNF-α + 1, per doubling	1.64	1.14–2.35	0.007
Leptin/adiponectin ratio, per doubling	1.41	0.98–2.02	0.066
Uric acid, per 1 mg/dL	1.65	1.16–2.35	0.005
Age, per year	0.97	0.91–1.03	0.265
Male sex	0.39	0.12–1.24	0.112
Type 2 diabetes	6.30	2.01–19.77	0.002
BMI, per kg/m^2^	0.98	0.88–1.09	0.697

## Data Availability

De-identified patient-level data supporting the findings are not publicly posted because of institutional privacy and ethics constraints. They may be made available by the corresponding authors upon reasonable request, subject to Ethics Council approval, institutional data-sharing policies, and applicable privacy requirements.

## Abbreviations

The following abbreviations are used in this manuscript:

AUC	Area under the receiver operating characteristic curve
BMI	Body mass index
CI	Confidence interval
DSM-BIA	Direct segmental multi-frequency bioelectrical impedance analysis
EIA	Enzyme immunoassay
GGT	Gamma-glutamyl transferase
HC3	Heteroscedasticity-consistent type 3 standard error
HDL-C	High-density lipoprotein cholesterol
HOMA-IR	Homeostasis Model Assessment of Insulin Resistance
IL-6	Interleukin-6
IQR	Interquartile range
IR	Insulin resistance
LDL-C	Low-density lipoprotein cholesterol
OR	Odds ratio
ROC	Receiver operating characteristic
SD	Standard deviation
STROBE	Strengthening the Reporting of Observational Studies in Epidemiology
T2DM	Type 2 diabetes mellitus
TNF-α	Tumor necrosis factor-alpha
